# Authenticity of claims made in drug promotional literature

**DOI:** 10.4103/0253-7613.62397

**Published:** 2010-02

**Authors:** Mangala Bhaskar Murthy, Bhaskar Krishnamurthy

**Affiliations:** Department of Pharmacology, GMC. Miraj, Flat. No.8, Mahalakshmi Complex, Pusphraj Chowk, Near Rohini Hotel, Sangli - 416416, Maharashtra, India. E-mail: mangala.bhaskar@gmail.com; 1 Department of Obstetrics and Gynecology, GMC. Miraj, Flat. No.8, Mahalakshmi Complex, Pusphraj Chowk, Near Rohini Hotel, Sangli - 416416, Maharashtra, India

Sir,

Misleading drug advertising encourages drug consumerism rather than rational use of drugs. To add to the problem, developing countries lack a strong system to keep a check on such activities.[1,2] We have analyzed the authenticity of information provided in the form of promotional literature in India. The study was conducted in Sangli (Maharashtra). Promotional literature for the study was collected from general practitioners and specialists. These were exposed to a primary screening process and only those promoting modern (allopathic) medicine and making at least one therapeutic claim were included in the study. Authenticity of therapeutic claims made in promotional literature was verified by accessing standard literature through internet databases like Medline and Cochrane reviews, standard text books and peer reviewed journals. Subsequently, the claims were classified as-

1. Authentic - When they were in concordance with the known benefit of drug administration.
2. Exaggerated - When a claim extended beyond the actual benefit obtained by the patient following drug administration.
3. Controversial - When only a few clinical studies in standard literature supported claims made on promotional literature while others disposed them.
4. Misrepresentation of data from published authentic literature to suit the claims made by the company in promotional literature.
5. False - When there were no studies to support the use of the drug for a particular claim made in promotional literature.

A total of 134 promotional leaflets given to medical practitioners were collected. Out of these, 102 satisfied the inclusion criteria and were subjected to analysis. The analysis showed that as many as 20% of the claims were exaggerated, 32% were inconclusive, 17% were false and only 21% were authentic. In 10% of the leaflets, original data from literature was misrepresented to suit the therapeutic claims made. Since a complete report of all these claims is beyond the scope of this article, a few examples are shown in Table 1. The results of other studies conducted worldwide, especially in the developing countries are in concordance with the present study.[2,3] To conclude, there is still an unmet need for dispersal of unbiased information to the prescribers. We also conclude that the promotional literature continues to be far from educational. We emphasize the need to inculcate the art of critical appraisal amongst medical practitioners, so that they may derive the best from the information made available to them in the form of promotional literature.

**Table 1:** Analysis of therapeutic claims made in promotional literature

*Active ingredient(s)*	*Claims made*	*Actual value of the claim*	*Comments*
Calcium and vitamin D3	Correct vitamin D deficiency and help reduce cardiovascular risk.	Misrepresentation of data from standard literature.	Vitamin D deficiency is associated with incident cardiovascular diseases, but further studies are required to determine whether correction of deficiency could prevent them
Aceclofenac and serratiopeptidase	Bacterial biofilm can reduce effective concentration at the site of infection-------- In traumatic infections, abscess and swelling, increase antibiotic efficacy.	Misrepresentation of data from standard literature.	The study was conducted in animals and directly extrapolated to human beings. Evidence for serratiopeptidase increasing antibiotic efficacy in human beings is inconclusive.
Ondansetron	Nausea and vomiting of pregnancy—first choice always	Exaggerated claim	Ondansetron belongs to category B in pregnancy (safety not well established by randomized controlled trials in human beings). Pyridoxine and Doxylamine and not ondansetron are first choice drugs for treatment of nausea and vomiting during pregnancy.
Olmesartan	The only ARB providing double digit BP reduction.	Exaggerated claim	Double digit BP reduction can mean reduction ranging from 10-99%. Most anti hypertensive drugs produce double digit BP reduction.
R - Sibutramine	Give to all your overweight, obese patients.	Exaggerated claim	Drug treatment of obesity is not warranted in all obese, overweight patients but only in those with BMI of 27 or more with diabetes or patients with BMI > 30 kg/m.^2^
Folic acid and docosahexaenoic acid.	For better gestational health and wiser child	Controversial claim	There is no conclusive evidence based on long term studies to support the use of docosahexaenoic acid during pregnancy to improve child wisdom.
Micronized progesterone	Preventing spontaneous deliveries	Controversial claim	Supplementation of progesterone to prevent preterm deliveries is based on the fact that preterm deliveries are a result of progesterone withdrawal. But there are many reasons for preterm delivery including incompetent cervix, which cannot be corrected by progesterone supplementation.
Doxylamine, pyridoxine, folic acid.	No adverse effect reported	False claim	Doxylamine is a known sedative.
Cefixime, clavulanic acid.	Combats bacterial resistance, ensures victory.	False claim	Cefixime is resistant to gram negative betalactamases with poor gram positive activity. Addition of Clavulanic acid does not fight resistance.
